# Low-*ω*3 Fatty Acid and Soy Protein Attenuate Alcohol-Induced Fatty Liver and Injury by Regulating the Opposing Lipid Oxidation and Lipogenic Signaling Pathways

**DOI:** 10.1155/2016/1840513

**Published:** 2016-12-18

**Authors:** Karina Reyes-Gordillo, Ruchi Shah, Ravi Varatharajalu, Mamatha Garige, Leslie C. Leckey, M. Raj Lakshman

**Affiliations:** Lipid Research Laboratory, VA Medical Center and Department of Biochemistry and Molecular Medicine, The George Washington University, Washington, DC, USA

## Abstract

Chronic ethanol-induced downregulation of peroxisome proliferator-activated receptor gamma coactivator 1-alpha (PGC1*α*) and upregulation of peroxisome proliferator-activated receptor gamma coactivator 1-beta (PGC1*β*) affect hepatic lipid oxidation and lipogenesis, respectively, leading to fatty liver injury. Low-*ω*3 fatty acid (Low-*ω*3FA) that primarily regulates PGC1*α* and soy protein (SP) that seems to have its major regulatory effect on PGC1*β* were evaluated for their protective effects against ethanol-induced hepatosteatosis in rats fed with Lieber-deCarli control or ethanol liquid diets with high or low *ω*3FA fish oil and soy protein. Low-*ω*3FA and SP opposed the actions of chronic ethanol by reducing serum and liver lipids with concomitant decreased fatty liver. They also prevented the downregulation of hepatic Sirtuin 1 (SIRT1) and PGC1*α* and their target fatty acid oxidation pathway genes and attenuated the upregulation of hepatic PGC1*β* and sterol regulatory element-binding protein 1c (SREBP1c) and their target lipogenic pathway genes* via* the phosphorylation of 5′ adenosine monophosphate-activated protein kinase (AMPK). Thus, these two novel modulators attenuate ethanol-induced hepatosteatosis and consequent liver injury potentially by regulating the two opposing lipid oxidation and lipogenic pathways.

## 1. Introduction

Alcohol liver disease is a major cause of morbidity and mortality, affecting millions world-wide [1]. Long-term exposure of ethanol causes fatty liver disease or hepatosteatosis [2], which further leads to steatohepatitis, fibrosis, and finally cirrhosis that may result in death [3]. Hepatosteatosis is characterized by the accumulation of lipids, triglyceride and cholesterol, due to an imbalance between hepatic lipid degradation and synthesis, leading to an enlarged fatty liver [3]. Studies have shown that alcohol causes the following: (i) increased mobilization of adipose fat into the liver, due to increased adipose lipoprotein lipase, (ii) decreased fat oxidation due to downregulation of fatty acid oxidation genes, (iii) increased fat synthesis due to upregulation of lipogenic genes, and (iv) impaired synthesis of apolipoprotein B and secretion of very low density lipoprotein (VLDL), the major lipoprotein for the export of hepatic lipids to peripheral tissues [4].

Transcriptional coactivators peroxisome proliferator receptor coactivator 1 alpha (PGC1*α*) and peroxisome proliferator receptor coactivator 1 beta (PGC1*β*) as well as sterol regulatory element-binding proteins (SREBPs) play vital roles in regulating the lipid oxidizing and lipogenic genes and thereby control the progression of hepatosteatosis and the consequent onset of fibrosis and other forms of liver injury [5, 6]. Peroxisome proliferator-activated receptors (PPARs) are members of the nuclear hormone receptor super family that are ligand-dependent transcription factors. There are three isotypes, namely, PPAR*α*, PPAR*β*, and PPAR*γ*. Whereas PPAR*α* is expressed in all tissues controlling the fatty acid oxidation pathway genes, PPAR*γ* is primarily expressed in adipose tissue and the liver, regulating the lipogenic pathway genes. PPAR*β* is found in many tissues although mainly in gut, kidney, and heart [7–9]. It is linked to colon cancer [10] but has not been well studied. PGC1*α* regulates lipid oxidation pathway genes* via* PPAR*α* and PGC1*β* regulates lipogenic pathway genes* via* the sterol regulatory element-binding proteins SREB1a, SREB1c, and SREBP2 [11]. SREB1c predominantly regulates fatty acid biosynthesis while SREB1a and SREBP2 control cholesterol synthesis [3]. AMP activated protein kinase (AMPK) is known to be activated by phosphorylation to form phosphorylated AMPK (pAMPK), which, in turn, phosphorylates and inactivates acetyl CoA carboxylase (ACC) and the rate-limiting enzyme of lipogenesis [4, 12, 13]. PGC1*α* is controlled by silence regulator gene 1 (SIRT1), the eukaryotic equivalent of SIR2 gene in prokaryotes, and histone acetyltransferases (HAT) [14]. SIRT1 activates PGC1*α* by deacetylation while HAT inactivates PGC1*α* by acetylation [15]. On the other hand, SIRT1 destabilizes SREBP1c by deacetylation while HAT stabilizes SREBP1c by acetylation [16]. PGC1*β* is upregulated by dietary saturated fat and coactivates SREBP1c and liver X receptor (LXR) families of transcription factors leading to increased lipogenesis, lipoprotein transport, and VLDL secretion [17, 18]. Therefore, any modulator that can either activate PGC1*α via* the interplay between SIRT1 and histone acetyltransferase (HAT) or inactivate PGC1*β*/SREBP1c should be beneficial in preventing alcoholic hepatosteatosis and consequent liver injury.

Omega-3/6 fatty acids are polyunsaturated fatty acids (PUFA) obtained from fish and plant sources. The most common omega-3 PUFA are eicosapentaenoic acid (EPA), docosahexaenoic acid (DHA), and alpha-linolenic acid (ALA). Whereas algae and oils from fish such as salmon, mackerel, and herring are rich in EPA and DHA, ALA is found in vegetable oils such as canola, flax seed oil, soybean oil, and nuts such as walnuts [19]. Soy proteins (SP) are found in soybean legume containing all 8 essential amino acids and very low saturated fat [20]. In recent times, both omega-3 PUFA and SP have received increased attention due to their beneficial effects against cardiovascular disease, obesity, type 2 diabetes, and certain cancers, among others [19, 21, 22]. Low omega 3 fatty acids (low-*ω*3FA) are known to have lipid lowering effects in humans [23] while SP lowers plasma and liver cholesterol and triglycerides in both animals and humans [24]. Studies have shown that SP prevents hyperinsulinemia and reduces the expression of LXRs and SREBP1c mRNAs in obese Zucker rat model [25–27]. However, the molecular mechanisms by which these dietary modulators can control the two transcriptional coactivators are yet to be explored. In this study, we demonstrate the novel actions of low-*ω*3FA and SP in inhibiting alcoholic hepatosteatosis by regulating two opposing vital pathway genes of lipid degradation and synthesis* via* PGC1*α* and PGC1*β*, respectively. Therefore, low-*ω*3FA and SP are potentially potent dietary modulators that seem to have these profound lipid lowering properties involving lipid catabolic and anabolic pathways. Moreover, low-*ω*3FA and SP stimulate AMPK phosphorylation and block ethanol-induced increased lipogenesis. Thus, this may be the first time a systematic approach is made to alleviate alcoholic hepatosteatosis by the combined effects of novel natural modulators that promise to intervene with both lipid oxidizing and lipogenic pathways.

## 2. Material and Methods

### 2.1. Animals

Wild-type (WT) female Wistar rats (~150 g body weight) from Charles River, Wilmington, MA, were housed in pairs per cage in plastic cages, in a temperature-controlled room, at 25°C with 12-hours light-dark cycle. All animals were fed a pelleted commercial diet (Purina Rodent Chow, number 500, TMI Nutrition, St. Louis, MO) during the first week of acclimation period after arrival. Experiments were performed according to the approved institutional animal care and use committee protocol. Female rats were randomly divided into 4 groups of 5 rats each and were pair-fed Lieber-DeCarli control or ethanol (EtOH) liquid diets (36% total fat calories) with high-*ω*3FA (14.1% of calories as *ω*3FA) or low-*ω*3FA (2.7% of calories as *ω*3FA) fish oil or EtOH with SP for 4 weeks.

### 2.2. Diets

The diets are isocaloric and their formulations are according to the modified method of Lieber and DeCarli [28] with the recommended normal nutrients, vitamins, and minerals according to AIN-93 diet [29]. Thus, 36% of the total energy of ethanol diet is from fat, 20% from protein, 36% from EtOH, and the rest from the carbohydrate. The corresponding isocaloric control diet has isoenergetic amounts of dextrin-maltose in place of EtOH. EtOH concentration in the liquid diet was gradually increased starting at 1% level on day 1 and reaching the 5% level over a 7-day period to allow the animals to adapt to EtOH in the diet. These diets are supplemented with 120 IU of tocopherol/L and 200 mg/L of tertiary-butyl hydroquinone as antioxidants as per AIN-93 diet recommendations [28, 29].

### 2.3. Lipid and Lipoprotein Analysis

Blood samples were collected and centrifuged at 3100 rpm using a Beckman J6M (Beckman Coulter, Indianapolis, IN) for 10 min at 4°C. Separated serum, plasma, and liver samples were frozen at −80°C until assayed. Liver lipids and high density lipoproteins (HDL) were extracted as previously described [30, 31]. Cholesterol was analyzed using Sigma diagnostic kit number 352 (Sigma-Aldrich, St. Louis, MO) according to the method of Allain et al. [32] and triglycerides were analyzed using Sigma diagnostic kit number 339 (Sigma-Aldrich, St. Louis, MO) according to the method of McGowan et al. [33]. All protein concentration determinations were done according to Bradford method [34] with bovine serum albumin (BSA) as the standard.

### 2.4. Isolation of Plasma HDL and Its Labeling with [^3^H] Cholesteryl Oleate

HDL was isolated from various pooled groups of rat plasma according to Gidez et al. [30]. Protein concentration was determined colorimetrically using bovine serum albumin (BSA) as a standard [34]. HDL cholesterol content was measured according to Zlatkis and Zak [35]. HDL labeling with [^3^H] cholesteryl oleate was performed according to Basu et al. [36], and the specific activity is expressed as dpm/mg HDL cholesterol.

### 2.5. Quantification of Hepatosteatosis by Oil Red O

Livers from various experimental groups were cut into small pieces and washed immediately with ice cold PBS and mounted on optimum cutting temperature (OCT) embedding compound in peel-a-way embedding molds (Electron Microscope Sciences, Hatfield, PA). Liver tissues were cryosectioned and stained with oil red O to measure accumulation of lipid using an automated histometric system (Image-Pro Plus 6.1, Media Cybernetics, Bethesda, MD) as described previously [37]. The data are expressed as average oil red O percentage area of lipid staining. Values are means ± SEM.

### 2.6. RNA Isolation and Real-Time RT-PCR

The total RNA was isolated from each liver using the Tri-Reagent (Molecular Research Center, Cincinnati, OH) as manufacturer's instructions. Isolated total RNA was reverse transcribed by in vitro transcription as described by the manufacturer (Invitrogen, Carlsbad, CA). Quantitative real-time PCR was performed using a Bio-Rad iCycler using the SYBR green PCR mix (Bio-Rad, Hercules, CA). Typical real-time PCR reaction mixture included same amount of cDNA templates from RT, 10 pM of each primers, 10 *μ*M of dNTPs, 3 mM of MgCl_2_, 10x buffer, and 2 *μ* of high fidelity Taq DNA polymerase in a reaction volume of 50 *μ*L with 0.1x SYBR Green I. The PCR conditions were 3 min at 95°C followed by 40 cycles at 95°C for 30 seconds, 55°C for 30 seconds, and 72°C for 1 min. Each primer pair was first tested by regular PCR to be highly effective and specific for amplification. *β*-Actin was used as the standard housekeeping gene. Ratios of specific mRNA and actin mRNA expression levels were calculated by subtracting the threshold cycle number (Ct) of the target gene from the Ct of actin and raising 2 to the power of this difference. Ct values were defined as the number of PCR cycles at which the fluorescent signal during the PCR reaches a fixed threshold. Target gene expressions were expressed relative to *β*-actin expression. The various primer pairs for indicated rat genes and transcription factors are listed in Supplemental Table 1 in Supplementary Material available online at http://dx.doi.org/10.1155/2016/1840513.

### 2.7. Western Blot Analysis

Liver extracts from each experimental group were diluted into SDS-PAGE sample buffer [50 mM Tris (pH 6.8), 2% SDS, 10% glycerol, 15 mM 2-mercaptoethanol, and 0.25% bromophenol blue] and electrophoretically resolved in Novex (Life Technologies, San Diego, CA) 4–20% denaturing polyacrylamide gels. Proteins are electrophoretically transferred to PVDF membrane and processed for immunodetection using the corresponding polyclonal primary antibodies for each of the above factors. After thorough washing, the primary antibody was detected with horse radish peroxidase conjugated secondary antibody specific to IgG of the respective primary antibody. Protein bands were visualized by chemiluminescence and quantified using FluorChem Imager (Alpha Innotech, CA). The nuclear extracts from each group were analyzed for the level of SIRT1, PGC1*α*, and PGC1*β* and the mature form of SREBP1c in the respective groups using the respective specific antibodies, while total protein extracts were analyzed for the levels of ACC, c-Met, AMPK, and pAMPK using respective specific antibodies. To determine the levels of acetylated-PGC1*α*, the liver nuclear extract from each group was initially immunoprecipitated with anti-PGC1*α* followed by immunoblotting with acetylated lysine antibody. The polyclonal antibodies for all the above transcription factors were purchased from Santa Cruz Biotechnology (Santa Cruz, CA), Cayman Chemicals (Ann Arbor, MI), and UpState Cell Signaling Solutions (Lake Placid, NY). The specificity of each antibody was verified before use for the above analyses.

### 2.8. Immunoprecipitation Analysis

Immunoprecipitation was performed as previously described [38]. To determine the levels of acetylated-PGC1*α*, the liver nuclear extract from each group was initially immunoprecipitated with anti-PGC1*α* (Abcam, Cambridge, MA), followed by immunoblotting with acetylated lysine antibody (Cell Signaling Technology, Danvers, MA).

### 2.9. Statistical Analysis

Experimental data were statistically analyzed, employing the paired and unpaired “*t*” tests on the control and the experimental values. The appropriate data were analyzed by one-way or two-way analysis of variance (ANOVA) at *p* < 0.05 followed by Tukey contrast to evaluate the true correlation between various parameters.

## 3. Results

### 3.1. Effects of Chronic Ethanol, Low-*ω*3FA, or SP on Serum and Liver Lipids and Hepatic Lipid Score

**Figure 1:**
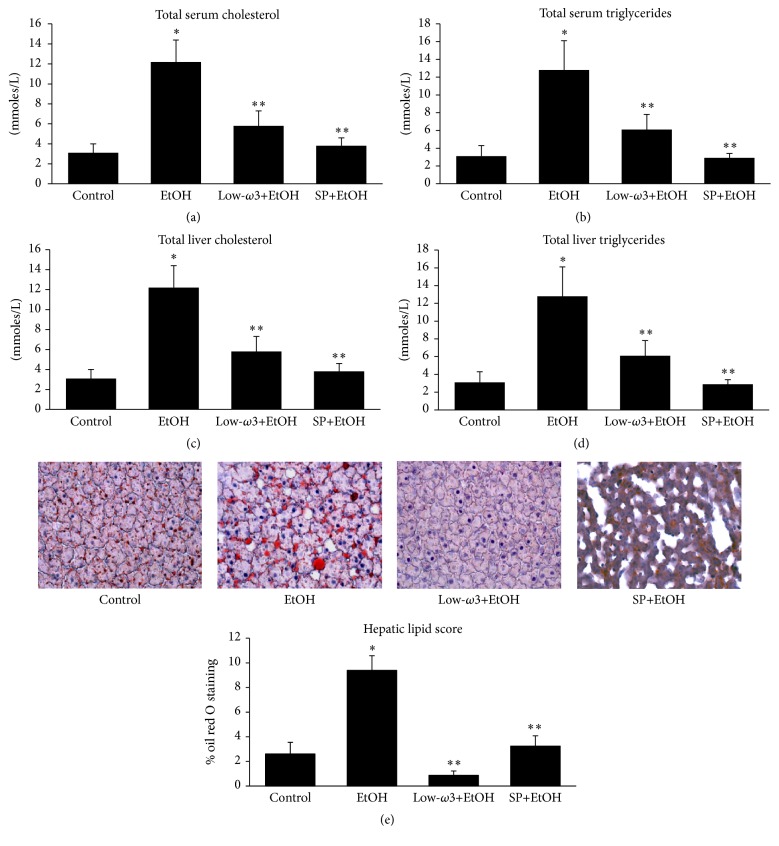
Influence of EtOH, low-*ω*3FA, and SP on (a) total serum cholesterol, (b) serum triglycerides, (c) total liver cholesterol, and (d) liver triglycerides. Each value is mean ± SD of 3 samples/group. (e) shows the representative medium-power (20x) photomicrographs of liver sections stained with oil red O as described in Section 2 and the plot of the mean hepatic lipid scores of all samples in each group ± SE of 3 samples/group. ^*∗*^ *p* < 0.05 versus control; ^*∗∗*^ *p* < 0.05 versus EtOH.

Serum cholesterol (Figure 1(a)) and triglycerides (Figure 1(b)) were significantly increased in EtOH group by 1.8-fold (*p* < 0.05) and 1.2-fold (*p* < 0.05), respectively, compared to control. Similarly, total liver cholesterol (Figure 1(c)) and triglycerides (Figure 1(d)) were also markedly increased in EtOH group by 3.9-fold (*p* < 0.05) and 4.1-fold (*p* < 0.05), respectively, compared to control. In contrast, dietary low-*ω*3FA or SP feeding to EtOH-fed groups significantly decreased serum and liver cholesterol and triglycerides to the level closer to that of the control group. Furthermore, the hepatic accumulation of lipids as measured by oil red O staining is markedly increased in EtOH group by 7.5-fold (*p* < 0.001) as compared to the control. This effect is significantly reduced after dietary administration of low-*ω*3FA and SP in the EtOH-fed group by 93% (*p* < 0.05) and 45% (*p* < 0.05), respectively (Figure 1(e)).

### 3.2. Effects of Low-*ω*3FA and SP on EtOH-Mediated Alterations in the Lipid Oxidation Pathway

**Figure 2:**
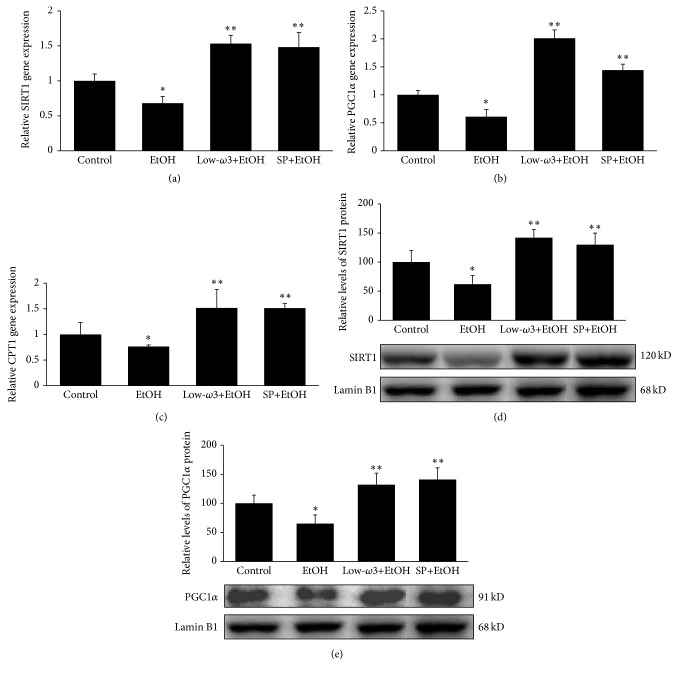
Influence of EtOH, low-*ω*3FA, and SP on lipid oxidation pathway. Total RNA from each animal was reverse transcribed and used in the qRT-PCR analysis using gene specific primers as described in Section 2 for (a) SIRT1, (b) PGC1*α*, and (c) CPT1. Each gene was normalized to *β*-actin mRNA. Nuclear protein was extracted from each animal and used for Western Blot analysis using specific antibodies as described in Section 2 for (d) SIRT1 and (e) PGC1*α*. Values are means of triplicate experiments ± SD of 3 samples/group and were corrected for difference in loading after reprobing with an antibody to Lamin B1. ^*∗*^ *p* < 0.05 versus control; ^*∗∗*^ *p* < 0.05 versus EtOH.

Chronic EtOH leads to a significant decrease in fatty acid oxidation (48.7 ± 5.8 nmoles/g/h, *p* < 0.05) as compared to control (100 ± 8.6 nmoles/g/h). We further investigated whether the mechanisms of action of low-*ω*3FA and SP on EtOH-induced decrease in fatty acid oxidation are mediated* via* the regulation of the transcriptional coactivator PGC1*α*, SIRT1, and the downstream pathway.  Figure 2(a) showed that low-*ω*3FA and SP treatment restored chronic EtOH-mediated 32% (*p* < 0.05) downregulation in SIRT1 mRNA by 85% (*p* < 0.05) and 80% (*p* < 0.05), respectively, as compared to EtOH group. EtOH also significantly downregulated PGC1*α* mRNA by 40% (*p* < 0.05) that was restored to 1.5-fold (*p* < 0.05) and 2-fold (*p* < 0.05) over the control level by low-*ω*3FA and SP treatment, respectively (Figure 2(b)). Additionally, CPT1 mRNA was also markedly downregulated by chronic EtOH (24%, *p* < 0.05) which was restored to 1.5-fold (*p* < 0.05) over the control level by these dietary modulators (Figure 2(c)). Similarly, chronic EtOH markedly decreased the nuclear protein expression of SIRT1 and PGC1*α* by 38% (*p* < 0.05) and 35% (*p* < 0.05), respectively, which was restored over the control levels by low-*ω*3FA and SP treatment (Figures 2(d) and 2(e)). PPAR*α*, a ligand-activated transcription factor, involved in the regulation of hepatic fatty acid oxidation [39], was also evaluated. EtOH significantly decreased PPAR*α* protein levels by 50% (*p* < 0.05) that was restored by 1.8-fold (*p* < 0.05) and 1.6-fold (*p* < 0.05) by low-*ω*3FA and SP treatment, respectively (see supplementary materials, Figure S1). Thus, low-*ω*3FA and SP are effective modulators in correcting the decreased fatty acid oxidation caused by chronic EtOH* via* the regulation of SIRT1, PGC1*α*, CPT1, and PPAR*α*.

**Figure 3:**
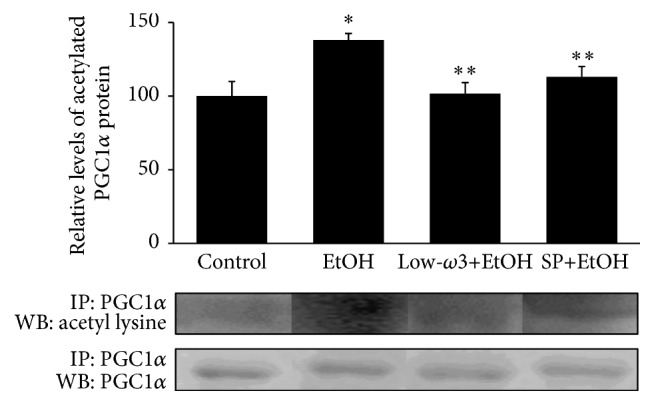
Effect of EtOH, low-*ω*3FA, and SP on hepatic acetylated-PGC1*α* levels. Western blot analysis was performed with an acetyl lysine-specific antibody of a nuclear protein extract immunoprecipitated (IP) with an antibody to PGC1*α* as described in Section 2. Values are means of triplicate experiments ± SD of 3 samples/group. ^*∗*^ *p* < 0.05 versus control; ^*∗∗*^ *p* < 0.05 versus EtOH.

In order to test whether the action of low-*ω*3FA and SP on hepatic lipid catabolism was mediated through the active or inactive forms of PGC1*α via* the modulation of SIRT1, we determined the level of acetylated PGC1*α* in the liver tissue of various groups.  Figure 3 shows that chronic EtOH increased the hepatic acetylated (inactive) form of PGC1*α* by 40% (*p* < 0.05) because of EtOH-mediated decrease in SIRT1 by 38% (*p* < 0.05) as compared to the control (Figure 2(d)), thereby accounting for decreased fatty acid oxidation. In contrast, low-*ω*3FA and SP decreased the inactive form of PGC1*α* by 37% and 25%, respectively, as compared to EtOH group (Figure 3)* via* the upregulation of SIRT1 (Figures 2(a) and 2(d)), thereby accounting for restoring the decreased fatty acid caused by chronic EtOH to the control level. Thus, low-*ω*3FA and SP may lower alcoholic hepatosteatosis by augmenting the relative levels of active form of PGC1*α*; that in turn effectively restored hepatic lipid catabolism that is impaired by chronic alcohol exposure.

### 3.3. Effects of Low-*ω*3FA and SP on Chronic EtOH-Mediated Alterations in the Lipogenic Pathway

**Figure 4:**
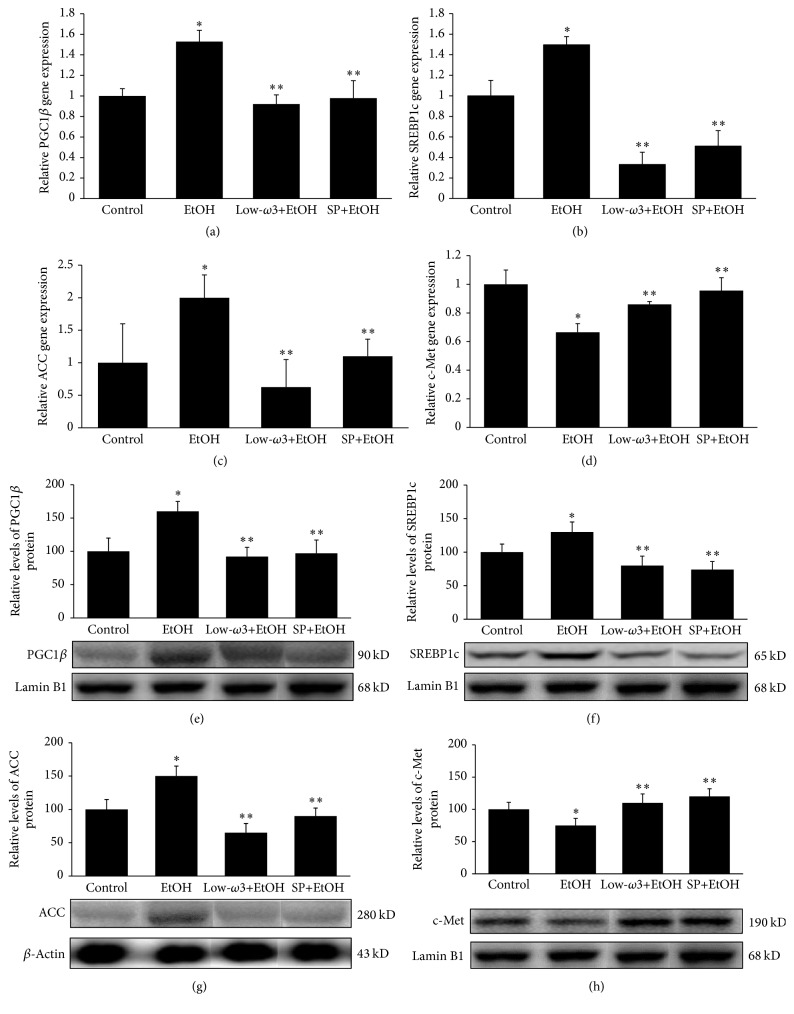
Influence of EtOH, low-*ω*3FA, and SP on lipogenic pathway. Total RNA from each animal was reverse transcribed and used in the qRT-PCR analysis using gene specific primers as described in Section 2 for (a) PGC1*β*, (b) SREBP1c, (c) ACC, and (d) c-Met. Each gene was normalized to *β*-actin mRNA. Nuclear or total protein was extracted from each animal and used for Western Blot analysis using specific antibodies as described in Section 2 for (e) PGC1*β*, (f) SREBP1c, (g) ACC, and (h) c-Met. Values are means of triplicate experiments ± SD of 3 samples/group and were corrected for difference in loading after reprobing with an antibody to Lamin B1 or *β*-actin for nuclear or total protein, respectively. ^*∗*^ *p* < 0.05 versus control; ^*∗∗*^ *p* < 0.05 versus EtOH.

Figure 4(a) shows that chronic EtOH markedly upregulated PGC1*β* mRNA level by 52% (*p* < 0.05) as compared to the control, and low-*ω*3FA and SP downregulated the EtOH effect by 61% (*p* < 0.05) and 55% (*p* < 0.05), respectively. Similarly, Figure 4(b) shows a marked 50% (*p* < 0.05) upregulation in SREBP1c mRNA that was reduced to 30% and 50% (*p* < 0.02) of the control value by low-*ω*3FA and SP treatment, respectively. Chronic EtOH also markedly upregulated the mRNA expression levels of ACC, which regulates fatty acid synthesis by 2-fold (*p* < 0.05) and this was significantly suppressed by 50% (*p* < 0.05) in the low-*ω*3FA-EtOH group and by 70% (*p* < 0.05) in SP-EtOH group (Figure 4(c)). In contrast, as shown in Figure 4(d), the mRNA expression levels of c-Met were significantly downregulated by 35% (*p* < 0.05) after chronic EtOH administration, and low-*ω*3FA and SP treatment significantly restored EtOH-induced downregulation of c-Met mRNA level to 86% (*p* < 0.05) and 95% (*p* < 0.05), of the control value, respectively. These results were confirmed by measuring the nuclear or total protein expression of the above genes relative to those of the corresponding subcellular marker proteins.  Figure 4(e) shows that low-*ω*3FA and SP fed rats showed suppressed EtOH-mediated increase (60%, *p* < 0.05) in the relative nuclear expression of PGC1*β* by 68% (*p* < 0.05) and 63% (*p* < 0.05), respectively. Similarly, as shown in Figures 4(f) and 4(g), the relative nuclear protein expressions of SREBP1c and ACC were also markedly increased in chronic EtOH group by 30% (*p* < 0.05) and 50% (*p* < 0.05), respectively, compared to the control group. Administration of dietary low-*ω*3FA and SP reversed these EtOH-mediated effects by decreasing SREBP1c protein expression by 50% (*p* < 0.05) and 56% (*p* < 0.05), respectively (Figure 4(f)), and ACC protein expression by 85% (*p* < 0.05) and 60% (*p* < 0.05), respectively (Figure 4(g)). On the other hand, c-Met expression was decreased in the EtOH group by 25% (*p* < 0.05), which were restored in low-*ω*3FA and SP groups by 35% (*p* < 0.05) and 45% (*p* < 0.05), respectively, as compared to the EtOH group (Figure 4(h)).

**Figure 5:**
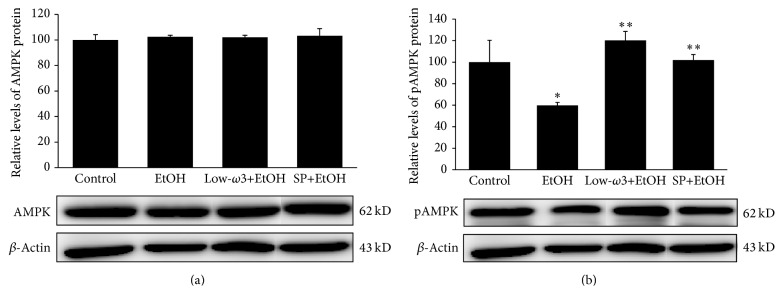
Effect of EtOH, low-*ω*3FA, and SP on hepatic (a) AMPK and (b) pAMPK was determined. Total protein was extracted from each animal and used for Western Blot analysis using specific antibodies as described in Section 2. Values are means of triplicate experiments ± SD of 3 samples/group and were corrected for difference in loading after reprobing with an antibody to *β*-actin. ^*∗*^ *p* < 0.05 versus control; ^*∗∗*^ *p* < 0.05 versus EtOH.

Since chronic EtOH increases hepatic ACC activity and lipogenesis by decreasing the phosphorylation of AMPK (pAMPK), a known inhibitor of ACC, we tested whether low-*ω*3FA or SP can counteract these effects of chronic EtOH by modulating the phosphorylation status of AMPK. As shown in Figures 5(a) and 5(b), although the level of total AMPK was unaffected in all groups, low-*ω*3FA and SP restored the hepatic level of pAMPK that was decreased by 50% (*p* < 0.05) in EtOH group. This increase in pAMPK could also account for decreased ACC activity and lipogenesis after low-*ω*3FA or SP treatment.

These findings are consistent with the ability of low-*ω*3FA or SP to (i) inhibit chronic EtOH-induced increase in lipogenic pathway genes and (ii) restore ethanol-mediated decreased intracellular transport of hepatic triglycerides to the blood compartment due to impaired VLDL assembly and secretion. This would lead to the low-*ω*3FA or SP-mediated reduction in fatty liver caused by chronic alcohol abuse.

## 4. Discussion

Our results show that low-*ω*3FA and SP exert their hypolipidemic action by upregulating primarily the lipid oxidizing genes* via* SIRT1 and PGC1*α* signaling pathway that are suppressed by chronic ethanol and downregulating the lipogenic pathway genes predominantly* via* the PGC1*β* and SREBP1c signaling pathway. Our data also support the alternative possibility that low-*ω*3FA and SP could prevent alcohol-induced activation of ACC activity by phosphorylating it* via* pAMPK.

SIRT1 is an NAD-dependent deacetylase (histone deacetylase (HDAC)) that has been linked to many beneficial effects of cellular processes including gene silencing, insulin resistance, glucose homeostasis, fatty acid metabolism, and aging, while HAT catalyses the opposite reaction [40]. Thus, SIRT1 activates PGC1*α* by deacetylation while HAT inactivates PGC1*α* by acetylation. On the other hand, SIRT1 destabilizes SREBP1c by deacetylation while HAT stabilizes SREBP1c by acetylation. You et al. [16] and Lieber et al. [41] have elegantly shown that both long chain and medium chain saturated fatty acids in the diet restore the expressions of SIRT1 and PGC1*α* that are downregulated by long chain polyunsaturated fatty acids (PUFA) in chronic ethanol-fed animals. However, PPAR*γ* was unaffected by chronic ethanol. Previously, Fischer et al. [42] have shown in mice that ethanol leads to PPAR*α* dysfunction resulting in impaired fatty acid oxidation and consequent onset of fatty liver that is overcome by a PPAR*α* agonist. Similarly, other studies [43, 44] have shown that alcohol-mediated fatty liver and injury are prevented by PPAR*γ* agonist presumably by activating c-Met and blocking alcohol-mediated induction of TNF*α*. We recently showed that compared to high fish oil control liquid diet, feeding of the same high fish oil liquid diet containing 5% (w/v) ethanol for 8 weeks significantly downregulated hepatic SIRT1, and PGC1*α* with the concomitant decreased hepatic rate of fatty acid oxidation [37]. Nanji et al. [45], Ronis et al. [46], and Song et al. [47] have demonstrated that saturated fatty acids protect against chronic alcohol-induced liver injury as compared to high levels of polyunsaturated fatty acids. In addition, Huang et al. [48] demonstrated that low levels of omega 3 polyunsaturated fatty acids, mainly docosahexaenoic acid, suppressed ethanol-induced hepatic steatosis. Similarly, Wada et al. [49] also demonstrated that low levels of fish oil fed prior to ethanol administration prevent acute ethanol-induced fatty liver in mice. In agreement with these studies, the present study shows that dietary low level of *ω*3FA (2.7%), but not high level of *ω*3FA (14.1%), restores the expression of SIRT1 and PGC1*α* that are downregulated by chronic ethanol. Our results also show that chronic alcohol exposure upregulates PGC1*β*, ACC, c-Met, and SREBP1c. Activation of SREBP1c by ethanol feeding in rats had been already associated with increased expression of hepatic lipogenic genes as well as the accumulation of triglyceride in the livers [50]. However, Ki et al. [51], Lu et al. [52], and Zeng et al. [53] demonstrated that SREBP-1c-mediated lipogenesis pathway was not affected by ethanol or even suppressed after chronic ethanol intake in rats. While the inability of these studies to show chronic ethanol-mediated upregulation of SREBP-1c-mediated lipogenic pathway may be due to different dietary fat compositions compared to the present study, we consistently find that the upregulation of this pathway with the high-*ω*3FA diet was markedly attenuated by low-*ω*3FA diet. Thus, it is important to point out that different dietary conditions, particularly the amount and the type fat in the diet, do affect SREBP activation pathway in different ways.

Low-*ω*3FA have an inherent property of attenuating chronic alcohol-mediated hepatosteatosis by upregulating PGC1*α* and downstream lipid degradation pathways while SP downregulates PGC1*β*, SREBP1, and downstream lipid synthetic pathways and by controlling the active/inactive forms of AMPK. Recently, Phillipson et al. demonstrated the lipid lowering effects of fish oil rich in *ω*3FA in humans [23]. However, chronic ethanol-induced liver damage in rats fed a high fat diet (36% fat calories) was exacerbated [45, 46, 54–56] with polyunsaturated FA from either vegetable oil (*ω*6 family) or fish oil (*ω*3 family) as evidenced by increased serum aspartate aminotransferase and alanine aminotransferase as well as by histopathology. In this study, fish oil constituted 36% of the total calories in the diet, which amounted to 14.1% of the total dietary calories as *ω*3FA. In contrast, we showed [57] that the inclusion of only 2.7% of total dietary calories as *ω*3FA resulted in lower plasma and liver lipids in chronic alcohol-fed animals. Furthermore, the same low level of dietary *ω*3FA restored the decreased ApoE content in HDL. Thus, a low level of *ω*3FA has beneficial effects [58–60], whereas a significant increase in *ω*3FA seems to have a detrimental effect on the liver [45, 46, 54–56]. It is possible that increased ethanol consumption in the intragastric model could have also caused the deleterious effects when PUFA-rich diet was fed [55]. Significantly, PUFA-containing lecithin diet was shown to prevent alcohol-induced hepatic fibrosis in baboons [61]. We showed [57] that low-*ω*3FA caused decreased VLDL production and serum lipids resulting in lipid-deficient ApoE, which can be easily sialylated and be associated with HDL. This would be consistent with the effects of low-*ω*3FA in reversing ethanol-mediated decrease in HDL-ApoE. Our previous work [58] also demonstrated that HDL from low-3FA-fed animals were more efficient in carrying out reverse cholesterol transport (RCT) function compared to the control animals regardless of whether the animals were on alcohol or control diet. We found [62] that cholesterol uptake by Hep-G2 cells from reconstituted HDL was stimulated by sphingomyelin (SPM). HDL phospholipid acyl chain composition is known to influence cholesterol efflux [63]. We also showed that chronic ethanol preferentially decreased SPM concentration in HDL of alcoholics leading to its impaired RCT function [64].

The present study shows that SP downregulated ethanol-mediated overexpression of PGC1*β*, SREBP-1, and its target lipogenic genes such as ACC (Figure 2), whereas it restored ethanol-mediated downregulation of SIRT1, PGC1*α*, and lipid oxidizing genes such as CPT1 (Figure 4). Overall, our results suggest that the relative hypolipidemic effects of SP compared to low-*ω*3FA in regulating alcoholic hepatosteatosis were more due to alteration in the lipogenic pathway, whereas that of low-*ω*3FA compared to SP was more due to alteration in the lipid oxidizing pathway.

In summary, this study has demonstrated the following. (1) Low-*ω*3FA and SP reduced alcoholic hyperlipidemia as well as hepatic lipid accumulation as evidenced by decreased liver cholesterol and triglycerides as well as hepatic histological lipid scores. (2) Low-*ω*3FA and SP prevented alcohol-mediated downregulation of SIRT1 and PGC1*α* and their target fatty acid oxidation pathway genes. (3) Low-*ω*3FA and SP attenuated alcohol-mediated upregulation of PGC1*β*, SREBP1c, and its target lipogenic pathway genes. (4) Low-*ω*3FA and SP decreased the liver nuclear SREBP1c level that was increased by chronic ethanol treatment. (5) Low-*ω*3FA and SP restored the hepatic level of pAMPK that was decreased by chronic alcohol treatment.

## 5. Conclusion

Unlike high dietary *ω*3FA, low dietary *ω*3FA protects against chronic alcohol-induced liver injury. We have demonstrated that low-*ω*3FA and SP could potentially upregulate SIRT1/PGC1*α* and downregulate PGC1*β*/SREBP1c signaling pathways in alleviating alcoholic hepatosteatosis and liver injury. Thus, our study opens this field to explore other new therapeutic agents targeted on PGC1*α* and PGC1*β* pathways for protection against not only alcoholic liver diseases but also metabolic syndrome and obesity, the major world-wide health problems, especially when superimposed in alcohol abusers.

## Supplementary Material

The Supplementary Material contains the list of primer sequences used for RT-PCR in this study and the Western blot analysis of PPAR alpha.
