# Supplementary material for: Low-ω3 Fatty Acid and Soy Protein Attenuate Alcohol-Induced Fatty Liver and Injury by Regulating the Opposing Lipid Oxidation and Lipogenic Signaling Pathways
Source: Oxid Med Cell Longev. 2016 Dec 18;2016:1840513. doi: 10.1155/2016/1840513 (PMC5203909; doi:10.1155/2016/1840513)
Supplement: Supplementary file 1 — The Supplementary Material contains the list of primer sequences used for RT-PCR in this study and the Western blot analysis of PPAR alpha. [file 1840513.f1.pdf]

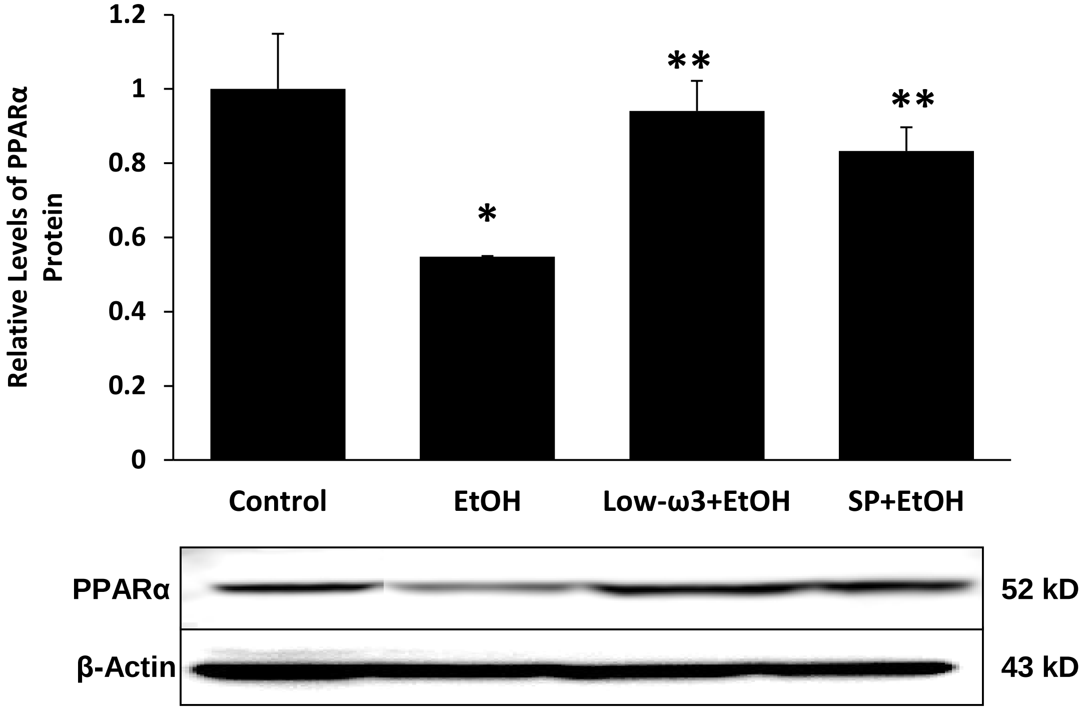

Relative Levels of PPARα Protein
1.2
1
0.8
0.6
0.4
0.2
0
*
**
**
Control
EtOH
Low-ω3+EtOH
SP+EtOH
PPARα
52 kD
β-Actin
43 kD

SUPPLEMENTAL TABLE
Supplemental Table 1: Rat RT-PCR Primer Sequences

Gene	Primer Sequence	
PGC1β	Forward	5’ – ctggctcctcatcctgtagc – 3’
	Reverse	5’ – ccggagagatttcggatgta – 3’
SREBP1c	Forward	5’ – ggagccatggattgcacatt – 3’
	Reverse	5’ – aggaaggcttccagagagga – 3’
cMet	Forward	5’ – cgacattcagtccgaggttca – 3’
	Reverse	5’ – gggacactggcctgactcttc – 3’
ACC	Forward	5’ – gcctcttcctgacaaacgag – 3’
	Reverse	5’ – gccgaaacatctctctgggata – 3’
SIRT1	Forward	5’ – cctttcagaaccaccaaagc – 3’
	Reverse	5’ – acagcaaggcgagcataaat – 3’
PGC1α	Forward	5’ – aacaagcacttcggtcatcc – 3’
	Reverse	5’ – agagcaagaaggcgacacat – 3’
CPT1	Forward	5’ – gtgaagcctttgggtggata – 3’
	Reverse	5’ – cagggtctcactcctcttgc – 3’
β-Actin	Forward	5’ – cagggtctcactcctcttgc – 3’
	Reverse	5’ – cagggtctcactcctcttgc – 3’
